# Health Risk Behavior Patterns in a National Adult Population Survey

**DOI:** 10.3390/ijerph15050873

**Published:** 2018-04-27

**Authors:** Ulrich John, Monika Hanke, Jennis Freyer-Adam

**Affiliations:** 1 Partner Site Greifswald, German Center for Cardiovascular Research (DZHK), Institute of Social Medicine and Prevention, University of Medicine Greifswald, Walther-Rathenau-Str. 48, D-17475 Greifswald, Germany; 2 Institute of Social Medicine and Prevention, University of Medicine Greifswald, Walther-Rathenau-Str. 48, D-17475 Greifswald, Germany; hanke@uni-greifswald.de; 3 Partner Site Greifswald, German Center for Cardiovascular Research (DZHK), Institute for Medical Psychology, University of Medicine Greifswald, Walther-Rathenau-Str. 48, D-17475 Greifswald, Germany; freyer@uni-greifswald.de

**Keywords:** health risk behaviors, smoking, alcohol risk drinking, overweight, inactivity, sex, age, education

## Abstract

*Background*: The aim of this paper is to analyze the co-occurrence of health risk behaviors (HRBs), namely, tobacco smoking, alcohol risk drinking, overeating, and physical inactivity, as well as their 16 combinations (patterns), which are stratified by age and gender. *Methods*: The data of 19,294 study participants, from a telephone survey among the adult general population of Germany that was conducted in 2012, were analyzed. *Results*: In adults, two or more of the four HBRs were found among 51.5% of females and 61.9% of males. The single most prevalent HRB pattern among all of the female (20.7, 19.6–21.8%) and male participants (18.2, 17.1–19.3%) was being overweight combined with a lack of physical activity, and its prevalence increased by 4% with each year of life. A multinomial regression analysis revealed that education was inversely associated with 11 of the 15 HRB patterns. The risk of having four, compared to zero, HRBs was 3.3 (2.5–4.4) for males relative to females. *Conclusion*: Similar to the findings from other western countries, the majority of the participants in this adult national sample from Germany had two or more HRBs. The most common of all possible HRB patterns was overweight and inactivity. The data confirm inverse relations between education and most HRB patterns.

## 1. Introduction

Common health risk behaviors (HRBs) include tobacco smoking, alcohol risk drinking, overeating, and physical inactivity. These four HRBs are particularly significant for morbidity and mortality. They are core factors for cardiovascular health, as defined by the American Heart Association [1], and for cancer prevention [2]. In many studies of preventive action, these HRBs have been analyzed as single factors, however they have not primarily been analyzed in interaction with each other. In recent years, considerable evidence for the number of HRBs has been established, that is, how many HRBs a person exerts at the same time or throughout their life [3]. Less is known about specific combinations of single HRBs, that is, the HRB patterns [4,5,6]. Discrepancies in HRB assessment and criteria impede on the comparison of findings between countries. HRB patterns provide the opportunity to make preventive interventions more efficacious, by addressing two or more behaviors that are particularly prevalent in a specified population. HRB patterns might help to prioritize preventive action.

Data has revealed that the number of HRBs is related to disease risk in a dose–response manner. According to general population data, the risk of death was lower if the number of HRBs was lower [7,8]. The prevalence of HRB patterns has been analyzed using data of the National Health and Nutrition Examination Survey (NHANES) of the U.S.A. [7]. Among women, the two most prevalent HRB patterns were at-risk alcohol consumption and physical inactivity (10.8%), as well as these two HRBs along with an unhealthy diet (12.9%), while the most prevalent HRB patterns among men were ever smoking and an unhealthy diet (10.2%), as well as these two HRBs along with physical inactivity (15.7%) [7].

General population data revealed associations between the level of education and HRBs. The proportion of those with the lowest degree of education had an increased number of HRBs [9]. In England, the subpopulation with the lowest social class had higher odds of one or more HRBs than those in the highest social class [10]. The data suggested a trend towards a dose–response relationship with those in the lowest social class, having an odds ratio of 2.4 (2.1–2.7) for three HRBs, compared with those in the highest social class [10]. The number of HRBs was also associated with the proportion of unemployed people [9]. In comparison to the general population, more HRBs were found among job-seekers between the ages of 18 and 64 years. The longer unemployment lasted, the higher the prevalence of three or more of the six HRBs [11].

One limitation of the research so far was the lack of data on the 16 possible combinations of the four HRBs, for different age groups and stratified by sex. According to age, the data suggest a trend towards the odds of having two or more, versus zero HRBs, being lower the older the study participants were [10]. The data revealed more HRBs for males than females [11,12], although physical inactivity was found to be more prevalent among female job-seekers than among male job-seekers [11]. Little is known about the associations between the level of education and specific HRB patterns.

We aimed to analyze the numbers and patterns of the four HRBs, stratified by sex and age groups, in a national telephone survey sample from Germany. With a focus on easy to define HRBs, data will first be used to describe the number and patterns of the HRBs for the total sample, stratified by sex and age. Secondly, the HRB numbers and patterns will be analyzed as a function of the level of education, adjusted for sex and age.

## 2. Materials and Methods

### 2.1. Sample

Data from the German Health Update (GEDA 2012) telephone survey were analyzed [13]. The data were provided by the National Health Monitoring Institute, who had also conducted the survey [14]. The sample was drawn according to the approach of random digit dialing [15]. The target population was the German population of 18 years old or older, who were living in a private household with a landline telephone number. A random sample of the potential telephone landline numbers was drawn. To account for the potential telephone numbers that were not included in the public registers, a procedure that was adapted from the U.S. standard was used [15]. In the second step, the private household numbers were identified. Thirdly, the target person per household was identified using a random selection procedure, in which all of the adult persons that were living in the private household were considered, based on the age of each household member [13]. Adults with a landline telephone number and a private residency in Germany, who were stratified by the seven socioeconomic areas of the nation, were eligible. The respondents were interviewed between February 2012 and March 2013 [13]. The response, that is, the number of realized interviews with complete data among all of the numbers that were likely to be household telephone numbers, according to the American Association for Public Opinion Research [16], was 22.1% [13]. The maximum number of contact attempts was 15. Among the target persons who were successfully contacted, 76.7% participated in the interview. The sample that was provided for the data analysis included 19,294 individuals.

### 2.2. Assessments

To assess the HRB numbers and patterns, four variables were used, namely, current tobacco smoking, alcohol risk drinking, being overweight, and physical inactivity. Each of the four variables were coded as 1 if they were present and 0 if they were absent, which resulted in an HRB number of 0 to 4. The current tobacco smoking was assessed according to the question, “Do you currently smoke? Please also consider occasional smoking”. The response categories were as follows, yes daily; yes, less than daily; not anymore; and never smoked.

Alcohol risk drinking was assessed by quantity and frequency questions, according to the Alcohol Use Disorders Identification Test-Consumption (AUDIT-C) [17]: “How often do you have an alcoholic drink, for example, a glass of wine, beer, mixed drink, spirits, or liquor?”. The response categories were as follows, 0: never, 1: monthly or less, 2: two to four times a month, 3: two to three times a week, 4: four times a week or more often. “How many drinks do you have on a typical day? I mean a small bottle of beer with 0.33 L, a small glass of wine with 0.125 L, a glass of sparkling wine, a double shot of spirits, or a bottle of alcopops” The response categories were as follows, 0: one to two drinks, 1: three to four drinks, 2: five to six drinks, 3: seven to nine drinks, and 4: ten or more drinks. “How often do you have six or more alcoholic drinks on one occasion, for instance at dinner or at a party?” The response categories were as follows, 0: never, 1: less than monthly, 2: monthly, 3: weekly, 4: daily or almost daily. A sum score from these three questions encompassed a range from 0 to 12. A score of 4 or more for females, and 5 or more for males, indicated alcohol risk drinking [18].

In our study, overeating was defined as a higher calorie intake than energy consumption, which was indicated by being overweight. Being overweight was estimated by the body mass index (BMI), which is defined as body weight in kilograms, divided by body height in square meters. Body weight and height were assessed by the questions, “How tall are you without shoes on, in centimeters?” and, “How much do you weigh without clothes on, in kilograms?” Being overweight was assumed if the BMI was 25.0 or higher.

Physical inactivity was assessed based on the questions, “For how many days a week are you physically active in a way that you start sweating or get short of breath?”, where the response categories were between 0 to 7 days a week, and, “How long on these days are you usually physically active?”, where the response categories were 60 min or more, 30 min to less than 60 min, 10 min to less than 30 min, and less than 10 min. In order to determine physical inactivity, the total amount of minutes per week were used, that is, the product of the number of days of physically activity and the number of minutes of physical activity per day. The minutes per day were calculated as the following, 60 min: 60 min or more, 45 min: between 30 min and 60 min, 20: between 10 min and 30 min, and 5: less than 10 min. Those with less than 150 min per week were subsumed to be physically inactive.

### 2.3. Statistical Analysis

The HRB patterns were the 16 combinations of the four HRBs that were analyzed. Age indicated age groups (18–29, 30–39, 40–49, 50–59, 60–69, 70–79, and 80 or older) in the descriptive analysis. The age group of 80 or older was considered as one group because only 65 individuals were 90 or older. In the regression analysis, age was used as a continuous variable. Education indicated 9 or less, 10, 11, and 12 or 13 years of a school education, according to the German school system. There were missing data for 796 individuals in the HRB sum score. For current smoking, alcohol risk drinking, being overweight, and physical inactivity, we replaced the missing values using the mean, which considered the seven age groups, sex, and school education. The missing values for school education were not replaced. There were 19,280 individuals left available for prevalence data analysis.

We calculated the prevalence rates. For the analysis of sex, age, and education, a multinomial logistics regression analysis was performed with the number and patterns of HRBs as dependent variables and no HRB was used as the reference group. Examining education, we controlled for sex and age. The estimated coefficients were transformed to relative risk ratios [19]. The data for mean, proportions, and regression analysis were used with weights, absolute numbers without weights. We utilized Stata version 14.1 with survey commands to account for the sampling weights and stratification by the primary sampling units. The sampling weights had been applied for sex, age, and education, based on the German national general population survey, in which participation was mandatory (microcensus) in 2011. To compare the means, we used *t*-tests.

## 3. Results

Among the sample of 19,294 individuals, the mean age was 59.9 (59.5–50.2) years, and 51.3% were female. Among females, for school education, 32.1% had 9 years or less, 37.7% had 10 years, 8.4% had 11 years, and 21.9% had 12 or 13 years. Among males, the respective proportions were 31.3%, 33.8%, 10.4%, and 24.5%, respectively.

Among females, 9.9% had no HBRs, 38.6% had one HBR, 50.2% had two or three HBRs, and 1.3% had four HRBs (Table 1). The mean number of HRBs was 1.54 (SD 0.88). According to the single health risk behaviors, the most prevalent HBR was inactivity (64.8% of all women), followed by being overweight (46.4% of all women), and then current smoking (24.0% of all women). Alcohol risk drinking occurred among 20.5% of all women. According to co-occurrences, being overweight plus inactivity were particularly prevalent (29.6% of all women). Being overweight plus inactivity plus smoking, was present in 5.5% of all women. The combinations of smoking plus alcohol risk drinking were present in 6.3% of all women. HRBs tended to be more prevalent among older rather than among younger female cohorts, although among the 18 to 29 year old females, 30.2% reported current smoking and 32.9% alcohol risk drinking, whereas among the 70 to 79 year old women, 8.1% reported current smoking and 17.9% alcohol risk drinking. Between the ages of 18 to 29 years, 20.3% were overweight and 62.3% were inactive. Between the ages of 70 to 79 years, 61.7% were overweight and 70.3% were inactive. Between the ages of 18 to 29 years, the proportion of overweight plus inactivity was 11.9% versus 43.5% among all females between the ages of 70 to 79.

Among all men, 8.2% had no HRBs, 29.9% had one HRB, 58.6% had two or three HRBs, and 3.3% had four HRBs (Table 2). The HRB mean number was 1.78 (SD 0.95). This was higher than the mean for women (*t*-test *p* < 0.001). The most prevalent HRB was being overweight (59.7% of all men), followed by physical inactivity (56.2% of all men), alcohol risk drinking (32.0% of all men), and current smoking (31.5% of all men). Being overweight plus inactivity (29.6% of all men) tended to be more prevalent among men than among women, as did being overweight combined with inactivity and smoking (9.0% of all men). The pattern of overweight plus inactivity plus alcohol risk drinking was present among 10.1% of all men. HRBs tended to be more prevalent in the older rather than in the younger male study participants. As was the case with women, the proportions of men with current smoking and of men with alcohol risk drinking tended to be lower among older compared to the younger study participants. Among males between the ages of 18 to 29, 38.5% reported current smoking and 41.3% reported alcohol risk drinking. Between the ages of 70 to 79, the respective figures were 13.5 and 29.4%. Among men between the ages of 18 to 29, a proportion of 31.7% reported being overweight and 42.1% reported physical inactivity. Between the ages of 70 to 79, 70.0% reported being overweight and 67.1% reported physical inactivity. Among all males between the ages of 18 to 29, the proportion of males who were overweight and inactive was 14.0% versus 46.1% among all males between the ages of 70 to 79. Compared to females (10.8%), males between the ages of 70 to 79 tended to include a particularly high proportion of individuals with three or four HRB (18.4%).

The level of education was found to be associated with risk for the number of HRBs and for HRB patterns, after adjustment for sex and age, and with study participants who had zero HRBs as the reference group (Table 3). It was found that the higher the education, the lower the risks for one, two, three, and four HRBs, as well as for 11 of the 15 HRB patterns. The relative risk to have four HRBs, compared to those with nine years of school education, was 0.8 (0.7–0.9). When considering the level of education, the relative risks were the most pronounced for current smoking, current smoking plus being overweight, current smoking plus being overweight plus alcohol risk drinking, and current smoking plus being overweight plus physical inactivity. The four relative risks were lower than 0.60. These four HRB patterns included current smoking, and three of them also included being overweight. The reference group for education showed the relative risk of 0.5 (0.5–0.6) for the pattern of smoking plus being overweight.

Education was unrelated to three of the HRB patterns. These included alcohol risk drinking, physical inactivity, and the combination of alcohol risk drinking and physical inactivity plus current smoking. Education was positively related to one HRB. It showed the relative risk of 1.1 (1.0–1.3) for alcohol risk drinking plus physical inactivity.

Men had a higher relative risk for two or more HRBs than women, and this relation increased by the number of HRBs. The risk ratio of four HRBs was 3.3 (2.5–4.4) for males compared with females. Men had a higher risk than women for 11 HRB patterns. The strongest relative risks, according to sex differences, were found for three HRB patterns, which all included alcohol risk drinking and being overweight. Compared with women, men had the relative risk of 5.9 (4.0–8.7) for current smoking combined with alcohol risk drinking and being overweight, and the relative risk of 3.3 (2.5–4.5) for having all four of the HRBs combined. There was no risk difference between men and women found for current smoking and current smoking plus physical inactivity. Males were at lower risk than females to be physically inactive (0.6; 0.5–0.7) and to be physically inactive combined with drinking alcohol in a risky way (0.8; 0.6–0.98).

The older the persons were, the more increased their risk of having one or more HRBs was, namely, of being overweight or inactive as single HRBs, of being overweight plus inactive, or of being overweight plus inactive combined with smoking or alcohol risk drinking. Six patterns, which included inactivity, were more likely among older subpopulations than among younger subpopulations. The relative risk of being overweight and inactive was 1.04 (1.04–1.05) for each year of life.

## 4. Discussion

Four main findings were revealed: Firstly, more than 50 per cent of the general adult population in Germany had two or more HRBs. Secondly, both among women and men, the most prevalent HRBs were being overweight and physical inactivity. Thirdly, the level of education was inversely associated with most of the HRB patterns. Fourthly, females were healthier than males, in terms of the number of HRBs.

The data clearly revealed that the majority of the adult population in Germany exhibited multiple health risk behaviors. Among the female and male respondents, 51.5% and 61.9%, respectively, had two or more of the four HRBs (all four HRBs: 1.3% and 3.3%, respectively); 38.6% and 29.9% had one, respectively; and 9.9% and 8.2%, respectively, had none of the four HRBs. This is in line with data from several other nations, including the United States and 11 European countries [9,10,12,20,21], according to which the majority of the adult population had two or more HRBs. According to the U.S. general population survey data, which included 16,958 study participants aged 17 or older (NHANES) [7], more than 75% of the participants had two or more of the four HRBs. While this proportion corresponded to the results that were found in our study for males in Germany, our results also supported those found in a cohort study from one area of Germany, which included 23,153 study participants between the ages of 35 to 65, which found that the majority (59.1%) had two or three HRBs, and 9.1% had none of the four HRBs [22]. Data from the U.S. National Health Interview Survey (NHIS) revealed comparable numbers, with 3.0% of the adults having four, 54.7% two or three, 32.6% one, and 9.7% none of the four HRBs [21].

The single most prevalent HRB pattern was being overweight combined with inactivity. The prevalence tended to be the higher the older the cohort was. Between the ages of 70 to 79, these two single health risk behaviors were present in more than 60% of the sample group. Being overweight combined with inactivity was reported in 43.5% of all females and in 46.1% of all males between the ages of 70 to 79. This finding, of the highest prevalence of HRBs, was in line with NHIS data from the United States in 2001 [21]. Unlike more recent U.S. samples, the proportions of (a) those who were overweight were lower, with 46.4% and 59.7% among the German females and males, respectively, compared with 55.4% and 69.0% among U.S. females and males, respectively; and (b) proportions of current smokers were higher, with 24.0% and 31.5% among the German females and males, respectively, compared with 18.0% and 22.4% among U.S. females and males, respectively [23].

The lower the level of education, the more likely 11 of the 15 HRB patterns were. Particularly strong associations were revealed for four HRB patterns. All four included smoking, three of them included the combination of smoking and being overweight, with the relative risk ratios being below 0.60. Current smoking and being overweight seemed to particularly affect populations with a low level of education. Our findings, according to the inverse relation between the level of education and HRB patterns, were in line with evidence regarding the associations of smoking and overweight with low life expectancy [24]. The higher the level of education, the more likely the population was to be free of any HRBs, compared with those who had one or more HRBs. In contrast to the findings from other countries [21,25], we did not find a dose–response relationship between the number of HRBs and education. Instead, our data suggested that a social gradient might have been apparent concerning whether or not any HRB was found.

The findings also spoke in favor of an inverse relation between education and alcohol-related HRB patterns [26]. Among the eight patterns, which included alcohol risk drinking, five were inversely associated and two were not associated with education. One HRB pattern, alcohol risk drinking combined with physical inactivity, was more likely the higher the level of school education. Alcohol risk drinking might have added to mortality, and a relationship between low socio-economic status and increased alcohol-attributable mortality risk existed [26].

Clearly, men had a higher number of HRBs than women. Three or four HRBs were present in 22.3% of all men and in 12.4% of all women. Compared with women, the data revealed a relative risk ratio of 3.3 for men for having four HRBs, versus no HRBs. The finding of more HRBs among men than among women was in line with former studies [20,21]. Men were more likely than women to have HRB patterns that included current smoking and alcohol risk drinking. The risk for males to currently smoke, drink alcohol in a risky way, and being overweight was almost six times as high as that for females. More men than women had patterns that included being overweight or being overweight combined with inactivity. There were only two patterns, physical inactivity or physical inactivity in conjunction with alcohol risk drinking, which were more likely among women than among men. This was in line with data from 11 European countries [25].

The older the participants were, the higher their risk of having one or more HRBs was, and of being overweight or inactive was. Other studies found that there were less HRBs present at age 65 or older, compared with those below the age of 65 [12,21], as well as a trend towards lower odds of having two or more HRBs compared with the youngest adults [10]. Our data suggested that the prevalence of the overweight plus inactivity pattern was 4% higher for each year of life, after adjustment for sex and education. Reduced physical activity as well as increased overweight might have been because of bodily complaints, lack of training, or the expectation that older people usually practice a more sedentary lifestyle than younger people. Tobacco smoking and alcohol risk drinking became less prevalent at an older age. This might have been as a result of quitting smoking and alcohol risk drinking, but also because of a selective mortality as a result of smoking or alcohol risk drinking and related health disturbances. Balancing out our findings about being overweight plus inactivity, and smoking plus alcohol, older populations did not seem to live healthier lives than younger populations.

Three limitations of our study should be mentioned. Firstly, the analyzed data underlay the restrictions of telephone health surveys in general. The proportion that took part in the survey was 22.1% of the target population. Findings might have been subject to selection bias, although the data was weighted. It may be assumed that the prevalence of the health risk behaviors and their patterns might have been even higher than what the data revealed. No data were available regarding the comparisons of respondents and non-respondents. Secondly, the assessment of physical inactivity did not consider different intensities, that is, moderate and vigorous, and different types of physical activity, that is, aerobic or muscle strengthening activities. Thirdly, BMI was assessed by self-statement only. The survey data had revealed that self-statements were likely to underestimate overweight [27]. Body weight included data from only 18,902 of the 19,294 study participants.

## 5. Conclusions

Among this national adult general population sample, the majority of the adult general population in Germany had two or three HRBs. The most common of all possible HRB patterns was being overweight combined with inactivity. The data confirm inverse relations between education and most of the HRB patterns. Females lived healthier than males, in terms of the number of HRBs. The older the population was, the higher the prevalence of one, two, or three HRBs was. Decreased smoking and alcohol risk drinking, but increased overweight and inactivity occurred with older age. These findings must be interpreted with caution, particularly because of selection bias that has to be assumed for this telephone survey sample. The implications of our study include that the findings provided a rationale for focusing preventive action on more than one health risk behavior, and for considering the variability of health risk behavior patterns by education and gender.

## Figures and Tables

**Table 1:** Multi-behavior risk constellations females, % (95% confidence interval).

HRB Pattern	Total	Age						
		18–29	30–39	40–49	50–59	60–69	70–79	80 or Older
*N*	9967	1220	1164	1827	1847	1712	1609	588
0 HRB	9.9 (9.2–10.7)	14.1 (11.9–16.6)	13.1(10.8–15.9)	11.2 (9.3–13.3)	7.3 (6.0–8.8)	9.4 (7.9–11.2)	7.5 (6.0–9.3)	2.9 (1.8–4.6)
Smoking	4.2 (3.6–4.7)	5.0 (3.8–6.6)	7.9 (6.0–10.4)	5.1 (4.0–6.5)	4.9 (3.7–6.5)	1.9 (1.2–3.1)	1.0 (0.4–2.2)	0.4 (0.1–2.6)
Alcohol risk drinking	3.1 (2.6–3.5)	7.2 (5.5–9.3)	1.7 (1.1–2.7)	2.7 (1.9–3.7)	3.1 (2.2–4.5)	2.3 (1.6–3.1)	2.5 (1.6–4.0)	0.7 (0.4–1.6)
Overweight	11.1 (10.2–12.0)	4.9 (3.5–7.0)	9.5 (7.3–12.2)	9.9 (8.0–12.2)	13.8 (11.6–16.3)	13.8 (11.7–16.3)	15.5 (13.1–18.3)	9.1 (5.8–13.9)
Inactivity	20.3 (19.3–21.4)	25.6 (22.8–28.7)	24.1 (21.3–27.1)	21.0 (18.7–23.5)	13.8 (12.0–15.8)	14.4 (12.1–17.1)	18.9 (16.3–21.9)	29.8 (24.7–35.5)
1 HRB	38.6 (37.3–39.9)	42.7 (39.4–46.1)	43.2 (39.6–46.8)	38.7 (35.8–41.7)	35.6 (32.7–38.7)	32.4 (29.3–35.7)	38.0 (34.6–41.5)	40.0 (34.3–46.1)
Smoking plus alcohol risk drinking	1.4 (1.2–1.8)	3.0 (2.0–4.5)	2.0 (1.3–2.9)	1.9 (1.3–2.8)	1.4 (0.8–2.3)	0.6 (0.3–1.0)	0.3 (0.1–0.7)	-
Smoking plus inactivity	6.3 (5.7–7.0)	8.0 (6.3–10.1)	8.8 (6.8–11.3)	9.0 (7.3–11.1)	6.4 (5.1–8.0)	4.9 (3.4–6.9)	1.9 (1.2–2.9)	1.6 (0.7–3.6)
Smoking plus overweight	3.0 (2.5–3.5)	1.5 (0.9–2.3)	3.3 (2.1–5.1)	4.3 (3.0–6.0)	5.8 (4.3–7.7)	2.5 (1.7–3.7)	0.4 (0.2–0.8)	1.4 (0.3–6.8)
Alcohol risk drinking plus overweight	2.1 (1.7–2.5)	1.2 (0.7–1.9)	1.4 (0.8–2.5)	1.2 (0.7–1.9)	3.8 (2.6–5.5)	2.7 (2.0–3.7)	2.3 (1.4–3.7)	2.1 (1.0–4.4)
Alcohol risk drinking plus inactivity	5.6 (5.0–6.2)	8.4 (6.8–10.3)	4.0 (2.9–5.4)	5.7 (4.6–7.0)	4.5 (3.5–5.8)	6.5 (5.1–8.4)	5.3 (3.9–7.3)	4.4 (2.4–8.0)
Overweight plus inactivity	20.7 (19.6–21.8)	6.2 (4.7–8.1)	13.3 (11.0–15.9)	15.2 (13.1–17.5)	19.9 (17.3–22.8)	29.0 (25.9–32.4)	33.5 (30.2–37.0)	41.9 (36.1–48.0)
2 HRBs	39.1 (37.8–48.4)	28.3 (25.3–31.4)	32.7 (29.3–36.2)	37.2 (34.3–40.2)	41.8 (38.6–45.0)	46.3 (42.8–49.7)	43.8 (40.3–47.3)	51.4 (45.3–57.4)
Smoking plus alcohol risk drinking plus overweight	0.6 (0.5–0.9)	0.8 (0.4–1.9)	1.0 (0.5–2.2)	0.6 (0.3–1.1)	1.1 (0.6–2.0)	0.4 (0.2–1.0)	0.0 (0.0–0.2)	0.0 (0.0–0.3)
Smoking plus alcohol risk drinking plus inactivity	3.0 (2.6–3.4)	8.4 (6.7–10.4)	2.9 (2.0–4.2)	2.9 (2.1–3.9)	2.5 (1.8–3.4)	1.8 (1.2–2.7)	0.7 (0.3–1.7)	0.2 (0.1–0.7)
Smoking plus overweight plus inactivity	4.2 (3.6–4.7)	1.8 (1.1–2.9)	4.2 (3.0–6.0)	5.9 (4.5–7.7)	5.9 (4.6–7.6)	4.5 (3.2–6.3)	3.2 (2.3–4.5)	1.1 (0.4–2.8)
Alcohol risk drinking plus overweight plus inactivity	3.4 (2.9–3.9)	2.2 (1.4–3.6)	1.5 (0.9–2.5)	2.3 (1.6–3.3)	3.8 (2.7–5.3)	4.3 (3.3–5.7)	6.2 (4.6–8.4)	3.8 (2.3–6.3)
3 HRBs	11.1 (10.3–12.0)	13.2 (11.1–15.7)	9.6 (7.7–11.9)	11.7 (9.8–13.8)	13.3 (11.3–15.6)	11.0 (9.1–13.3)	10.2 (8.2–12.7)	5.2 (3.4–7.9)
Smoking plus alcohol risk drinking plus overweight plus inactivity	1.3 (1.0–1.5)	1.7 (1.1–2.7)	1.4 (0.8–2.4)	1.2 (0.7–1.9)	2.0 (1.4–2.8)	0.9 (0.5–1.7)	0.6 (0.2–1.2)	0.5 (0.1–1.7)

Notes: Weighted proportions; *N*—number of persons unweighted; general population of Germany telephone survey sample; survey conducted March 2012–March 2013; Health risk behavior (HRB) pattern of single health risk behaviors; no observations (-).

**Table 2:** Multi-behavior risk constellations males, % (95% confidence interval).

HRB Pattern	Total	Age						
		18–29	30–39	40–49	50–59	60–69	70–79	80 or Older
*N*	9313	1423	1078	1835	1742	1610	1324	301
0 HRB	8.2 (7.5–8.9)	16.6 (14.3–19.1)	8.4 (6.3–11.1)	7.5 (6.1–9.1)	5.3 (4.1–6.8)	5.0 (3.9–6.4)	5.4 (4.0–7.2)	4.5 (1.9–10.1)
Smoking	3.3 (2.8–3.8)	6.3 (4.8–8.2)	5.6 (3.9–7.9)	2.5 (1.8–3.5)	3.0 (2.1–4.3)	1.5 (0.8–2.5)	0.6 (0.3–1.2)	-
Alcohol risk drinking	3.8 (3.4–4.4)	9.7 (8.1–11.6)	2.2 (1.3–3.6)	3.0 (2.1–4.3)	1.6 (1.1–2.4)	3.1 (1.9–5.2)	3.0 (1.9–4.8)	2.3 (0.9–6.1)
Overweight	12.4 (11.6–13.3)	5.5 (4.2–7.2)	13.8 (11.3–16.7)	13.4 (11.5–15.6)	13.8 (11.7–16.2)	16.3 (14.0–18.9)	13.3 (11.1–15.9)	11.7 (6.8–19.2)
Inactivity	10.3 (9.6–11.2)	11.7 (9.6–14.0)	10.9 (8.8–13.4)	8.8 (7.4–10.4)	8.5 (7.1–10.3)	8.0 (6.5–9.9)	12.4 (10.0–15.4)	23.5 (17.5–30.7)
1 HRB	29.9 (28.6–31.1)	33.1 (30.1–36.3)	32.4 (28.9–36.2)	27.8 (25.2–30.4)	27.0 (24.3–29.8)	29.0 (25.9–32.2)	29.3 (25.9–33.0)	37.4 (29.8–45.7)
Smoking plus alcohol risk drinking	2.8 (2.4–3.2)	7.6 (6.2–9.4)	3.6 (2.5–5.1)	2.5 (1.7–3.6)	1.7 (1.0–2.7)	0.5 (0.2–1.2)	0.0 (0.0–0.2)	-
Smoking plus inactivity	4.9 (4.3–5.6)	5.6 (4.1–7.7)	5.8 (3.9–8.4)	6.2 (4.6–8.4)	5.4 (4.1–7.0)	3.4 (2.4–4.9)	2.4 (1.4–4.2)	1.0 (0.2–5.1)
Smoking plus overweight	5.0 (4.4–5.7)	4.4 (3.2–6.1)	6.4 (4.8–8.4)	6.9 (5.3–8.9)	5.9 (4.4–7.9)	3.4 (2.1–5.5)	2.5 (1.3–4.4)	0.5 (0.1–2.6)
Alcohol risk drinking plus overweight	5.2 (4.6–5.8)	3.7 (2.7–5.1)	4.0 (2.5–6.4)	4.6 (3.6–5.8)	5.9 (4.7–7.5)	7.0 (5.4–9.0)	7.2 (5.5–9.4)	1.9 (0.9–4.1)
Alcohol risk drinking plus inactivity	3.6 (3.1–4.1)	5.3 (4.2–6.8)	3.4 (2.2–5.4)	2.5 (1.8–3.6)	2.4 (1.7–3.4)	3.1 (2.2–4.3)	3.7 (2.4–5.7)	9.6 (4.7–18.5)
Overweight plus inactivity	18.2 (17.1–19.3)	6.4 (4.9–8.4)	11.8 (9.6–14.4)	17.8 (15.6–20.1)	19.4 (16.8–22.3)	24.3 (21.2–27.6)	31.1 (27.4–35.1)	35.4 (28.0–43.5)
2 HRBs	39.6 (38.2–41.0)	33.1 (30.1–36.3)	34.9 (31.2–38.8)	40.5 (37.5–43.5)	40.7 (37.5–44.0)	41.7 (38.2–45.3)	46.9 (42.9–51.0)	48.4 (40.2–56.8)
Smoking plus alcohol risk drinking plus overweight	3.1 (2.7–3.6)	4.1 (3.1–5.5)	5.8 (4.4–7.7)	3.5 (2.6–4.7)	2.6 (1.8–3.8)	1.8 (1.1–2.9)	0.9 (0.4–1.9)	-
Smoking plus alcohol risk drinking plus inactivity	3.4 (2.9–3.9)	5.5 (4.3–7.1)	3.7 (2.6–5.3)	2.9 (2.1–4.1)	2.4 (1.7–3.5)	3.5 (2.0–6.1)	2.5 (1.3–4.5)	0.1 (0.0–0.7)
Smoking plus overweight plus inactivity	5.7 (5.0–6.4)	2.2 (1.4–3.4)	6.6 (4.7–9.3)	7.3 (5.7–9.3)	9.0 (7.2–11.3)	5.7 (4.3–7.6)	2.9 (1.6–4.9)	2.8 (1.2–6.4)
Alcohol risk drinking plus overweight plus inactivity	6.8 (6.2–7.5)	2.6 (1.8–3.7)	4.3 (3.0–6.0)	6.6 (5.2–8.4)	7.9 (6.4–9.7)	10.7 (8.6–13.3)	10.4 (8.4–12.9)	6.1 (3.8–9.7)
3 HRBs	19.0 (17.9–20.1)	14.4 (12.3–16.7)	20.5 (17.5–23.9)	20.3 (17.9–23.0)	22.0 (19.4–24.9)	21.7 (18.7–25.1)	16.7 (13.9–19.9)	9.0 (5.9–13.4)
Smoking plus alcohol risk drinking plus overweight plus inactivity	3.3 (2.9–3.9)	2.8 (1.9–4.1)	3.8 (2.6–5.5)	4.0 (3.0–5.2)	5.0 (3.6–6.9)	2.6 (1.7–3.9)	1.7 (0.8–3.5)	0.7 (0.1–3.3)

Notes: Weighted proportions; *N*—number of persons unweighted; general population of Germany telephone survey sample; survey conducted March 2012–March 2013; HRB pattern of single health risk behaviors; no observations (-); smoking—current smoking.

**Table 3:** Associations between sex, age, level of education, and health risk behavior patterns.

Behavioral Risk	Relative Risk	*p*	95% Conf. Interval
0 HRB	Reference		
1 HRB			
education, reference: 9	0.85	0.00	0.80–0.90
sex, reference: female	0.97	0.70	0.85–1.12
age, reference: 18	1.01	0.00	1.01–1.02
2 HRBs			
education	0.76	0.00	0.71–0.80
sex	1.32	0.00	1.15–1.52
age	1.02	0.00	1.02–1.03
3 HRBs			
education	0.75	0.00	0.70–0.80
sex	2.16	0.00	1.84–2.54
age	1.01	0.00	1.01–1.02
4 HRBs			
education	0.81	0.00	0.72–0.91
sex	3.32	0.00	2.49–4.42
age	1.008	0.49	1.00–1.02
Smoking			
education	0.58	0.00	0.52–0.66
sex	0.93	0.55	0.73–1.18
age	0.98	0.00	0.97–0.98
Alcohol risk drinking			
education	1.02	0.64	0.92–1.14
sex	1.50	0.001	1.19–1.90
age	0.994	0.20	0.986–1.003
Overweight			
education	0.70	0.00	0.65–0.75
sex	1.51	0.00	1.27–1.79
age	1.03	0.00	1.02–1.03
Inactivity			
education	1.02	0.55	0.95–1.09
sex	0.63	0.00	0.54–0.74
age	1.02	0.00	1.015–1.025
Smoking plus alcohol risk drinking			
education	0.71	0.00	0.62–0.80
sex	2.23	0.00	1.68–2.96
age	0.96	0.00	0.95–0.97
Smoking plus inactivity			
education	0.65	0.00	0.58–0.72
sex	0.95	0.68	0.76–1.19
age	0.99	0.04	0.99–1.00
Smoking plus overweight			
education	0.52	0.00	0.46–0.59
sex	2.10	0.00	1.63–2.71
age	1.00	0.58	0.99–1.005
Alcohol risk drinking plus overweight			
education	0.83	0.00	0.75–0.92
sex	3.29	0.00	2.57–4.22
age	1.03	0.00	1.02–1.04
Alcohol risk drinking plus inactivity			
education	1.14	0.01	1.04–1.25
sex	0.78	0.03	0.63–0.98
age	1.02	0.00	1.01–1.03
Overweight plus inactivity			
education	0.75	0.00	0.70–0.80
sex	1.25	0.01	1.07–1.46
age	1.04	0.00	1.04–1.05
Smoking plus alcohol risk drinking plus being overweight			
education	0.57	0.00	0.50–0.65
sex	5.91	0.00	4.03–8.67
age	0.98	0.00	0.98–0.99
Smoking plus alcohol risk drinking plus inactivity			
education	0.92	0.18	0.82–1.04
sex	1.35	0.02	1.05–1.73
age	0.99	0.01	0.98–0.997
Smoking plus overweight plus inactivity			
education	0.59	0.00	0.53–0.66
sex	1.78	0.00	1.42–2.24
age	1.01	0.00	1.006–1.02
Alcohol risk drinking plus overweight plus inactivity			
education	0.87	0.00	0.79–0.94
sex	2.74	0.00	2.20–3.42
age	1.04	0.00	1.035–1.05
Smoking plus alcohol risk drinking plus overweight plus inactivity			
education	0.80	0.00	0.71–0.91
sex	3.35	0.00	2.51–4.46
age	1.01	0.06	1.00–1.02

Notes: Multinomial logistic regression analysis; survey method; general population of Germany telephone survey sample; survey conducted March 2012–March 2013; reference—reference group; 18—age 18; 9—9 years school education; sex—1: females, 2: males; age—age 18 to 99; education—9 years or less, 10, 11, and 12 or 13 years school education; HRB—health risk behavior; smoking—current smoking; inactivity—physical inactivity; *p*—statistical significance (*p* < 0.05).
